# Characteristics and genomic epidemiology of colistin-resistant Enterobacterales from farmers, swine, and hospitalized patients in Thailand, 2014–2017

**DOI:** 10.1186/s12879-023-08539-8

**Published:** 2023-08-28

**Authors:** Adhiratha Boonyasiri, Lauren M. Brinkac, Elita Jauneikaite, Richard C. White, Chris Greco, Chakkraphong Seenama, Teerawit Tangkoskul, Kevin Nguyen, Derrick E. Fouts, Visanu Thamlikitkul

**Affiliations:** 1 Faculty of Medicine Siriraj Hospital, Mahidol University, Salaya, Thailand; 2 NIHR Health Protection Research Unit in Healthcare Associated Infections and Antimicrobial Resistance, Imperial College London, London, UK; 3 J. Craig Venter Institute, Rockville, MD 20850 USA; 4 Noblis, Reston, VA 20191 USA; 5 Department of Infectious Disease Epidemiology, School of Public Health, Imperial College, London, UK; 6 Division of Infectious Diseases and Tropical Medicine, Department of Medicine, Faculty of Medicine Siriraj Hospital, Mahidol University, Bangkok, Thailand

**Keywords:** Carbapenemase-producing Enterobacterales, Colistin resistance, Genomic epidemiology, Swine

## Abstract

**Background:**

Colistin is one of the last resort therapeutic options for treating carbapenemase-producing Enterobacterales, which are resistant to a broad range of beta-lactam antibiotics. However, the increased use of colistin in clinical and livestock farming settings in Thailand and China, has led to the inevitable emergence of colistin resistance. To better understand the rise of colistin-resistant strains in each of these settings, we characterized colistin-resistant Enterobacterales isolated from farmers, swine, and hospitalized patients in Thailand.

**Methods:**

Enterobacterales were isolated from 149 stool samples or rectal swabs collected from farmers, pigs, and hospitalized patients in Thailand between November 2014–December 2017. Confirmed colistin-resistant isolates were sequenced. Genomic analyses included species identification, multilocus sequence typing, and detection of antimicrobial resistance determinants and plasmids.

**Results:**

The overall colistin-resistant Enterobacterales colonization rate was 26.2% (*n* = 39/149). The plasmid-mediated colistin-resistance gene (*mcr*) was detected in all 25 *Escherichia coli* isolates and 9 of 14 (64.3%) *Klebsiella* spp. isolates. Five novel *mcr* allelic variants were also identified: *mcr*-2.3, *mcr*-3.21, *mcr*-3.22, *mcr*-3.23, and *mcr*-3.24, that were only detected in *E. coli* and *Klebsiella* spp. isolates from farmed pigs.

**Conclusion:**

Our data confirmed the presence of colistin-resistance genes in combination with extended spectrum beta-lactamase genes in bacterial isolates from farmers, swine, and patients in Thailand. Differences between the colistin-resistance mechanisms of *Escherichia coli* and *Klebsiella pneumoniae* in hospitalized patients were observed, as expected. Additionally, we identified mobile colistin-resistance *mcr*-1.1 genes from swine and patient isolates belonging to plasmids of the same incompatibility group. This supported the possibility that horizontal transmission of bacterial strains or plasmid-mediated colistin-resistance genes occurs between humans and swine.

**Supplementary Information:**

The online version contains supplementary material available at 10.1186/s12879-023-08539-8.

## Introduction

Antimicrobial resistance is recognized as one of the most significant global health concerns [1]. Carbapenemase-producing Enterobacterales (CPE) are resistant to a broad range of beta-lactam antibiotics, including carbapenems [2], leaving limited treatment options to treat infections caused by CPE. In general, colistin is used as a monotherapy or in combination with other antibiotics to treat CPE infections [3]. Consequently, the increase in CPE infections has resulted in the increased use of colistin, with the inescapable risk of emerging colistin resistance [4]. Notably, because some countries, such as China and Thailand, have widely used colistin in livestock for the treatment and prevention of infection, the emergence of colistin resistance may have been additionally promoted by the inappropriate use of human antibiotics in farm animals [5, 6].

Colistin is a polypeptide antibiotic belonging to a group of polymyxins with broad-spectrum activity against Gram-negative bacteria. It binds to the lipopolysaccharide (LPS) of bacteria, resulting in the leakage of intracellular components from the cell membrane followed by bacterial death [7]. Before 2015, the most commonly reported mechanisms of colistin resistance were those mediated by chromosome-encoded mutations in the *pmr*AB and *pho*PQ genes or by *mgr*B gene inactivation leading to lipid A modification and subsequent interference in the colistin interaction [8]. In 2015, the first plasmid-mediated colistin resistance gene, *mcr*-1, was reported in China [4]. The protein encoded by *mcr*-1 functions by transferring a phosphoethanolamine residue to lipid A, altering its structure and thereby lowering its affinity for colistin [9]. To date, *mcr*-1-containing isolates have been detected for several Enterobacterales species found in food-chain production, humans, and the environment worldwide [10, 11]. According to one retrospective study in China, the *mcr*-1 gene was initially identified from *Escherichia coli* in chickens in the 1980s [12]. Recently, novel *mcr* family genes, *mcr*-2 to *mcr*-10, have been reported across a range of Enterobacterales [13]. The discovery of horizontal transfer of colistin resistance triggered concerns about the impact of colistin use on the spread of colistin resistance in the animal production industry, especially regarding swine [14]. In fact, a possible link between swine and farmers in terms of colistin-resistant *E. coli* after direct contact, i.e., horizontal transmission, was reported in Laos in 2012 [15]. These findings suggest the possible loss of colistin efficacy for the treatment of multidrug-resistant Gram-negative bacteria, such as CPE, in hospitalized patients. Thus, it is imperative to understand the genomic epidemiology between food-production animals and humans.

The aim of the present study was to determine the molecular epidemiology of *mcr*-mediated colistin resistance genes and their genetic environment in *Escherichia coli* and *Klebsiella pneumoniae* in farmers, livestock, and hospitalized patients in Thailand between 2014 and 2017.

## Methods

### Ethics statement

The study protocol was approved by the Siriraj Institutional Review Board (Si571/2015 and Si680/2016). Written informed consent was obtained from all patients. The data extracted from the medical records were de-identified to protect patients’ confidentiality. Ethical review and approval was not required for the animal study because all stool or rectal swab samples were submitted from farmers and pigs in industrial field to the diagnostic laboratory as the annual surveillance. All methods were carried out in accordance with relevant guidelines and regulations such as the Declaration of Helsinki, the Belmont Report, CIOMS guidelines, and ICH-GCP guidelines.

### Population and specimen collection

Stool samples were collected from 83 hospitalized patients who were admitted for treatment in the internal medicine wards of Siriraj Hospital, Bangkok between December 2015 and June 2017, and from 28 healthy food-production animal farm workers in a northern province. One rectal swab was taken from each of 38 randomly-chosen healthy pigs raised on the same farm at which the farmers worked. The stool or rectal swab samplings of pigs and farmers were collected and submitted to the diagnostic laboratory by the farmers working at their industrial farms as a routine annual surveillance. These samples were collected between 1 November 2014 and 31 December 2017. Stool samples and rectal swabs from patients, farmers and swine were inoculated onto MacConkey agar (BD, USA) and any bacterial colonies observed after incubation at 35 °C for 24 h were subcultured for colony purification. Bacteria species were identified using matrix-assisted laser desorption ionization time-of-flight (MALDI-TOF) on a Bruker Microflex LT/SH instrument (Bruker, USA) in accordance with the manufacturer’s protocol.

### Antimicrobial susceptibility testing

Antibiotic susceptibility was determined by disk diffusion method. The tested antibiotics were cefoxitin, ceftriaxone, ertapenem, imipenem, meropenem, gentamicin, amikacin, nalidixic acid, ciprofloxacin, tetracycline, erythromycin, piperacillin/tazobactam, cefoperazone/sulbactam and colistin (Thermo Fisher Scientific, USA). Colistin resistance was further confirmed by the broth microdilution method. The minimum inhibitory concentrations (MICs) of colistin were determined by the broth microdilution in cation-adjusted Mueller–Hinton II broth according to Clinical and Laboratory Standards Institute (CLSI) 2017 guidelines [16]. *E. coli* ATCC 25922 was used as a control and a range of colistin dilutions (Chem-Impex Int’l Inc., USA) between 0.25 mg/L and 128 mg/L were performed. Breakpoints of colistin susceptibility defined by European Committee on Antimicrobial Susceptibility Testing (EUCAST) [17] were used as follows: isolates with a colistin MIC ≤ 2 mg/L were categorised as susceptible, and those with a colistin MIC > 2 mg/L were categorised as resistant.

### DNA extraction and whole-genome sequencing

DNA was prepared using the Wizard® Genomic DNA Purification Kit (Promega, USA). Paired-end 150-basepair Nextera XT libraries of whole genomic DNA were sequenced on the Illumina NextSeq® 500 platform (Illumina, USA) with a target average coverage of 100-fold was performed on the 39 colistin-resistant Thailand isolates. Twelve *mcr*-positive isolates were selected for long-read sequencing using high molecular-weight genomic DNA and the rapid barcoding kit (SQK-RBK004) to prepare libraries, which then were sequenced on a MinION R9.4 flow cell (Oxford Nanopore, UK) with a target average coverage of 20-fold to improve the respective draft genome assemblies.

### Bioinformatics analyses

All raw reads were *de novo* assembled using SPAdes v3.1.1 [18]. A subset of 12 genomes that also had long reads were assembled combining Illumina and Oxford Nanopore sequencing reads using Unicycler v0.4.7 [19]. Hybrid assemblies were iteratively polished using Illumina reads with Pilon v1.22 [20]. All assembled genomes were assigned bacterial species based on an average nucleotide identity (ANI) by comparing assembled genomes to all-type strain genome assemblies in GenBank using MASH v.1.1.1 [21].

A whole genome alignment was inferred from single nucleotide polymorphisms (SNPs) identified by the Northern Arizona SNP Pipeline (NASP) v1.0.2 [22]. NASP output files were run through snpEff [23], which provided variant annotations and predicted the effect of the SNP on the gene’s protein product. Additionally, the resulting NASP alignment was run through Gubbins v.2.2.1 [24] to filter out the effects of recombination before building maximum-likelihood phylogenetic tree using RAxML v8.2.12 [25] under the GTRCAT model with 100 bootstrap replicates. The resulting tree was rendered with metadata annotated using the Interactive Tree of Life v4 (iTOL) [26].

To determine phylogenetic relationship between identified novel variants of MCR proteins and previously published MCR protein variants, we followed the proposal for assignment of allele numbers for *mcr*-genes [27] and selected representative protein sequences of MCR-1.1 through MCR-7.1 from GenBank; additionally including sequences of MCR-8.1 (NG_061399, [28]) and MCR-8.2 (AXU00196, [29]). MEGA7 [30] was used to generate a MCR protein phylogeny tree.

To identify chromosomal colistin resistance variants, the following method was used: colistin-susceptible *K. pneumoniae* MGH 78578 (RefSeq accession no. CP000647-CP000652) was used as a reference for variant identification. The amino acid sequences of designated targets were aligned using Clustal Omega [31]. Jalview [32] was used to visualise the alignments and identify substitutions. Subsequently, substitutions identified among all colistin-resistant isolates were filtered against those found in colistin-susceptible isolates to sort alterations unique to colistin-resistant isolates.

Multi locus sequence typing (MLST) for *K. pneumoniae* and *E. coli* genomes was determined using LOCUST [33] and the following MLST databases: for *K. pneumoniae* - Pasteur Institut (http://bigsdb.pasteur.fr/klebsiella/) and for *E. coli* - Achtman MLST schemes (https://enterobase.warwick.ac.uk/species/ecoli/download_7_gene). Beta-lactamase and *mcr* genes were identified using The Resistance Gene Identifier (RGI) [34], with default parameters from the Comprehensive Antibiotic Resistance Database (CARD) [35]. LOCUST was used to identify *mcr* gene variants, and novel allele variants were appropriately named [27]. Identification of plasmid replicons was done using PlasmidFinder v2.0.1 [36].

### Nucleotide sequence accession numbers

All sequencing reads were deposited in the SRA database under the project accession number PRJNA389557, https://www.ncbi.nlm.nih.gov/bioproject/?term=PRJNA389557. Relevant assemblies and *mcr*-genes have been deposited in GenBank under accession numbers as shown in Suppl. Tables S1 and S2).

## Results

### Description of colistin-resistant isolates

A total of 149 stool samples or rectal swab samples were collected from hospitalized patients (*n* = 83), healthy farm workers (*n* = 28) and swine (*n* = 38) between November 2014 and December 2017. The most prevalent bacteria found in these samples were *E. coli* (50.3%; *n* = 75/149) followed by *K. pneumoniae* (46.3%; *n* = 69/149) and *K. quasipneumoniae* subsp. *similipneumoniae* (3.4%; *n* = 5/149). A total of 39 (26.2%) bacterial isolates were identified as colistin-resistant and were further analyzed in detail in this study (Table 1). The prevalence of colistin resistant isolates was 12.1% (*n* = 10/83) in hospitalized patients, 10.7% (*n* = 3/28) in farmers and 68.4% (*n* = 26/38) in swine.

**Table 1:** Baseline characteristics and antimicrobial resistance profiles of 39 colistin-resistant isolates and plasmid replicons identified in 12 *mcr-*harboring isolates with complete plasmids

Strain	Bacterial species	ST	Host	Collection date (mm/yy)	Ceftriaxone AST	Ertapenem AST	Colistin MIC (mg/L)	*mcr* genes	Beta-lactamase (*bla*) genes	Plasmid type
CTRSIUE-6	*E. coli*	410	Patient	04/2016	R	S	8	*mcr*-3.5	*bla*_CTX−M−55_, *bla*_TEM−1_	
ECCTRPRTH01	*E. coli*	3054	Patient	01/2017	R	S	4	*mcr*-1.1	*bla*_CTX−M−55_, *bla*_TEM−1_	
ECCTRPRTH02	*E. coli*	3054	Patient	01/2017	R	S	4	*mcr*-1.1	*bla*_CTX−M−55_, *bla*_TEM−1_	IncX4(CP002895)
ECCTRPRTH03	*E. coli*	224	Patient	01/2017	R	S	4	*mcr*-1.1, *mcr*-3.5	*bla*_CTX−M−55_, *bla*_TEM−1_	IncFII (AY458016)
ECCTRPRTH04	*E. coli*	156	Patient	01/2017	R	S	8	*mcr*-1.1	*bla*_CTX−M−55_	
ECCTRSRTH02	*E. coli*	206	Swine	11/2016	R	S	8	*mcr*-1.1, *mcr*-3.2	*bla*_CTX−M−55_, *bla*_TEM−1_	
ECCTRSRTH03	*E. coli*	34	Swine	01/2016	R	S	4	*mcr*-1.1, *mcr*-3.5	*bla*_CTX−M−55_, *bla*_TEM−1_	IncI2 (KP347127) IncR (DQ449578), IncX1 (EU370913)
ECCTRSRTH04	*E. coli*	165	Swine	06/2016	R	S	4	*mcr*-1.1	*bla*_CTX−M−14_	
ECCTRSRTH05	*E. coli*	1602	Swine	09/2016	R	S	8	*mcr*-2.3, *mcr*-3.4	*bla*_CTX−M−14_	IncFIB (AP001918), IncY (K02380)
ECCTRSRTH06	*E. coli*	1040	Swine	06/2016	R	S	8	*mcr*-1.1, *mcr*-3.5	*bla*_CTX−M−14_, *bla*_TEM−1_	
ECCTRSRTH07	*E. coli*	5218	Swine	09/2016	R	S	4	*mcr*-3.4	*bla*_CTX−M−55_	
ECCTRSRTH08	*E. coli*	398	Swine	04/2016	R	S	8	*mcr*-1.1, *mcr*-3.1	*bla*_CTX−M−55_, *bla*_TEM−1_	IncX4 (CP002895)
ECCTRSRTH09	*E. coli*	206	Swine	10/2016	R	S	8	*mcr*-1.1, *mcr*-3.1	*bla*_CTX−M−55_	IncFII (AY458016), IncX4 (CP002895)
ECCTRSRTH10	*E. coli*	165	Swine	04/2017	R	S	8	*mcr*-1.1, *mcr*-3.1	*bla*_CTX−M−55_, *bla*_TEM−1_	
ECCTRSRTH11	*E. coli*	34	Swine	01/2016	R	S	4	*mcr*-1.1, *mcr-*3.5	*bla*_CTX−M−55_, *bla*_TEM−1_	
ECH + 04	*E. coli*	7626	Farmer	11/2014	R	S	16	*mcr*-1.1, *mcr*-3.5	*bla*_CTX−M−55_, *bla*_TEM−1_	
ECH + 05	*E. coli*	3045	Farmer	11/2014	R	S	4	*mcr*-1.1	*bla*_CTX−M−55_, *bla*_TEM−1_	
ECH + 09	*E. coli*	354	Farmer	11/2014	R	S	8	*mcr*-1.1, *mcr*-3.1	*bla*_CTX−M−55_, *bla*_TEM−1_	
ECSW + 04	*E. coli*	10	Swine	08/2015	R	S	8	*mcr*-3.2	*bla*_CTX−M−55_, *bla*_TEM−1_	
ECSW + 05	*E. coli*	4429	Swine	08/2015	R	S	8	*mcr*-3.5	*bla*_CTX−M−55_, *bla*_TEM−1_	
ECSW + 06	*E. coli*	48	Swine	08/2015	R	S	4	*mcr*-3.1	*bla*_CTX−M−14_, *bla*_TEM−1_	
ECSW + 07	*E. coli*	7625	Swine	08/2015	R	S	4	*mcr*-3.1	*bla*_CTX−M−55_	
ECSW + 08	*E. coli*	48	Swine	08/2015	R	S	16	*mcr*-3.1	*bla*_CTX−M−14_, *bla*_TEM−1_	
ECSW + 09	*E. coli*	10	Swine	08/2015	R	S	8	*mcr*-1.1, *mcr*-3.5	*bla*_CTX−M−14_, *bla*_TEM−1_	
ECSW + 12	*E. coli*	10	Swine	08/2015	R	S	8	*mcr*-1.1, *mcr*-3.24	*bla*_CTX−M−14_	
KPCTRPRTH01	*K. pneumoniae*	16	Patient	05/2016	R	R	32		*bla*_OXA−232_, *bla*_CTX−M−15_, *bla*_NDM−1_, *bla*_TEM−1_, *bla*_SHV−1_, *bla*_OXA−9_	
KPCTRPRTH02	*K. pneumoniae*	16	Patient	03/2017	R	R	64		*bla*_OXA−232_, *bla*_CTX−M−15_, *bla*_NDM−1_, *bla*_TEM−1_, *bla*_SHV−1_	
KPCTRSRTH01	*K. pneumoniae*	3541	Swine	01/2016	R	S	16	*mcr*-3.22	*bla*_CTX−M−14_, *bla*_SHV−1_	Unknown incompatibility group
KPCTRSRTH02	*K. pneumoniae*	17	Swine	11/2016	R	S	32	*mcr*-3.22	*bla*_CTX−M−14_, *bla*_SHV−11_	
KPCTRPRTH03	*K. pneumoniae*	231	Patient	01/2017	R	R	64		*bla*_OXA−232_, *bla*_CTX−M−15_, *bla*_TEM−1_, *bla*_SHV−1_	
KPCTRPRTH04	*K. pneumoniae*	16	Patient	04/2017	R	R	32		*bla*_OXA−232_, *bla*_CTX−M−15_, *bla*_NDM−1_, *bla*_SHV−1_	
KPCTRPRTH05	*K. pneumoniae*	16	Patient	04/2017	R	R	64		*bla*_OXA−232_, *bla*_CTX−M−15_, *bla*_NDM−1_, *bla*_TEM−1_, *bla*_SHV−1_, *bla*_OXA−9_	
KPCTRSRTH06	*K. pneumoniae*	3542	Swine	04/2016	R	S	8	*mcr*-3.21, *mcr*-3-fs	*bla*_CTX−M−63_, *bla*_TEM−1_, *bla*_SHV−29−like_	IncFIB (JN420336) IncFII (pMET) (EU383016)
KPCTRSRTH07	*K. pneumoniae*	1	Swine	04/2016	R	S	4	*mcr*-3.21	*bla*_CTX−M−14_, *bla*_CMY−2_, *bla*_SHV−1_	IncFII(pMET)(EU383016)
KPCTRSRTH08	*K. pneumoniae*	1867	Swine	11/2016	R	S	16	*mcr*-3.23	*bla*_CTX−M−14_, *bla*_SHV−60_, *bla*_TEM−1_	
KPCTRSRTH09	*K. pneumoniae*	15	Swine	12/2016	R	S	16	*mcr*-3.21	*bla*_SHV−28_	IncFII(pMET)(EU383016)
KPCTRSRTH03	*K. quasipneumoniae* subsp. *similipneumoniae*	3387	Swine	09/2016	R	S	8	*mcr*-3.22	*bla*_OKP−B−5_, *bla*_CTX−M−55_	
KPCTRSRTH04	*K. quasipneumoniae* subsp. *similipneumoniae*	1318	Swine	06/2016	R	S	8	*mcr*-3.21*	*bla*_OKP−B−6_, *bla*_CTX−M−63_, *bla*_TEM−1_	IncFII (pMET) (EU383016) (1st copy), IncHI1B (JN420336) (2nd copy)
KPCTRSRTH05	*K. quasipneumoniae* subsp. *similipneumoniae*	421	Swine	09/2016	R	S	16	*mcr*-3.22	*bla*_OKP−B−8_, *bla*_SHV−2_	Unknown incompatibility group

AST, Antimicrobial susceptibility testing; MIC, Minimum inhibitory concentration; S, Susceptible; R, Resistant; fs, frameshift; *, two copies

Seventy-five *E. coli* isolates were isolated from 29 swine, 23 patients and 23 farmers. Twenty five out of 75 *E. coli* isolates were colistin resistant (17 were from swine, 5 from patients and 3 from farmers) (Table 1). The prevalence of colistin-resistant *E. coli* was 58.6% (*n* = 17/29) in swine, 21.7% (*n* = 5/23) in patients and 13% (*n* = 3/23) in farmers, respectively. The 25 colistin-resistant *E. coli* isolates could be differentiated into 18 distinct sequence types (STs): ST10 (*n* = 3), ST34 (*n* = 2), and ST48 (*n* = 2), all of which belong to clonal complex 10 (CC10) and are frequently obtained from farmed animals, along with ST165 (*n* = 2), ST206 (*n* = 2), ST3054 (*n* = 2), and 12 other STs represented by single isolates (Table 1). No single *E. coli* genotype was shared between isolates collected from farmers, swine, and patients. Among the 25 colistin-resistant *E. coli* isolates, the colistin MIC range was 4–16 mg/L (Table 1). None of the *E. coli* isolates were resistant to ertapenem (Table 1).

The remaining 14 colistin-resistant isolates were *Klebsiella* spp., 11 were *K. pneumoniae* and three were *Klebsiella quasipneumoniae* subsp. *similipneumoniae* (Table 1). Five of the 11 colistin-resistant *K. pneumoniae* isolates were obtained from hospitalized patients, while the rest of the *K. pneumoniae* and *K. quasipneumoniae* subsp. *similipneumoniae* isolates were obtained from healthy swine; no colistin-resistant *Klebsiella* spp. were isolated from farmers (Table 1). Four out of five *K. pneumoniae* isolates from patients were ST16, which is known to be a widely-distributed disease-causing *K. pneumoniae* clone [37]. Among the 14 colistin-resistant *Klebsiella* spp. isolates, the colistin MIC range was 4–64 mg/L (Table 1). Notably, all five *K. pneumoniae* isolates from patients were also resistant to ertapenem (Table 1).

### Mechanisms of colistin resistance

The *mcr* gene was detected in 87.2% (*n* = 34/39) of the isolates resistant to colistin. All of the *E. coli* isolates (*n* = 25) and 64.3% (*n* = 9/14) of the *Klebsiella* spp. isolates had *mcr* gene(s) present. Among the *E. coli* isolates, *mcr*-3 was more common in swine (94.1%, *n* = 16/17) than in humans (37.5%, *n* = 3/8), whereas *mcr*-1 was more common in humans (87.5%, *n* = 7/8) than in swine (52.9%, *n* = 9/17). Interestingly, 50% of *E. coli* isolates (*n* = 13/25) were found to contain two *mcr* genes, with 12 isolates (9 from swine, 2 from farmers and 1 from patients) coharboring *mcr*-1 and *mcr*-3 and one isolate from swine coharboring *mcr*-2 and *mcr*-3 (Table 1). All nine *Klebsiella* spp. isolates carried the *mcr*-3 allelic variant were from swine (Table 1).

**Fig. 1:**
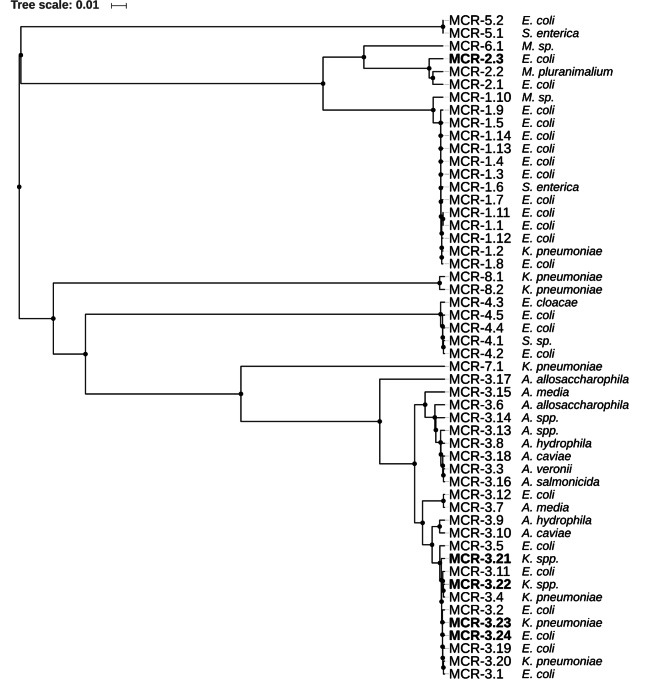
**Relationship between the sequences of publicly available and novel MCR proteins**. The UPGMA tree was constructed, using MUSCLE, from a full-length protein alignment of 47 publicly available and 5 novel *mcr* variants identified in this study. The scale represents the sum of mismatches over the total length of the aligned sequence

Five novel allelic variants of *mcr* were identified among the *mcr*-positive *E. coli* and *Klebsiella* spp. swine isolates: *mcr*-2.3 (Accession: NG_065452), *mcr*-3.21(Accession: QDJ85872), *mcr*-3.22 (Accession: QDJ80325), *mcr*-3.23 (Accession: NG_060583), and *mcr*-3.24 (Accession: NG_060580) (Fig. 1). A novel *mcr*-2 variant, *mcr*-2.3 found in *E. coli* ECCTRSRTH05, was 98.3% similar to a reported *mcr*-2 gene [38] with only nine amino acid changes in the encoded protein (Suppl. Table S3). Other four novel variants of *mcr*-3 were identified in both *E. coli* and *Klebsiella* spp., with an average 99.7% identity to a reported *mcr*-3 gene [39] with only one to two amino acid differences in the encoded protein (Suppl. Table S3). Four *Klebsiella* spp. isolates that had the same novel variant *mcr*-3.22 were isolated from swine, but they belonged to different genotypes (Table 1), excluding the possibility of clonal expansion. *E. coli* isolates carrying *mcr*-1.1 and/or variants of *mcr*-3 did not exhibit higher levels of MIC to colistin (MIC ranged from 4 to 16 µg/ml) when compared to isolates that carried only one copy of the mentioned *mcr* genes. This finding suggests that the co-occurrence of *mcr*-1.1 and variants of *mcr*-3 might not provide a synergistic or an additive effect on the colistin-resistant phenotype. Additionally, the coharboring of *mcr*-1.1 or variants of *mcr-3* and beta-lactamase (*bla*) genes was observed in the *E. coli* isolates from farmers and swine (Table 1). The most common *bla* genes observed in these isolates were *bla*_CTX−M−55_ and *bla*_TEM−1_ (Table 1).

Protein comparisons of the MCR protein (Fig. 1) showed the clustering of alleles based on their root gene; on average, the *mcr*-3 and *mcr*-2 alleles differed by an overall mean distance of 0.0345 and 0.01498, respectively, as computed using a Poisson model implemented in MEGA7 [30]. All other alleles had an average mean distance of 0.00529 within their clusters, indicating that *mcr*-3 is currently the most diverse *mcr* allele (Fig. 1). Notably, a second copy of *mcr*-3.21 was identified in KPCTRSRTH04 on a potential chromosomally encoded 2.8-Mbp contig, and KPCTRSRTH06 was found to contain a second copy of *mcr*-3 that is pseudogenized via a frameshift mutation, likely due to relaxed selection via the acquisition of a second *mcr*-3 copy (Table 1).

A diverse set of 12 plasmids containing *mcr*-1- and *mcr*-3 genes was identified in the *E. coli and K. pneumoniae* isolates (Table 1). The *mcr*-1.1 genes were predominantly found in isolates with IncX4 and IncI2 plasmids, while *mcr*-3 alleles were predominantly detected on IncFIB, IncFII, and IncR plasmids in swine isolates and the IncFII plasmid in *E. coli* isolated from hospitalized patients. Interestingly, the *mcr*-2.3 gene of ECCTRSRTH05 was identified on a potential chromosomally-encoded 2.7-Mbp contig. The IncFII (pMET) plasmid (EU383016) harboring *mcr*-3.21 was identified in four *Klebsiella* spp. isolates (Table 1). The *mcr*-3.22-containing plasmid in isolates KPCTRSRTH01 and KPCTRSRTH05 belonged to an unknown incompatibility group, with KPCTRSRTH01 being most similar to the *mcr*-3.11-containing IncR plasmid (MH341574.1) reported from China. Notably, two copies of *mcr*-3.21 were identified in KPCTRSRTH04. The first copy was located on the IncFII (pMET) plasmid (EU383016) and the second copy was located on a 2.8 Mbp contig, indicating that the second copy was chromosomally encoded (Table 1). The *mcr*-3 pseudogene was identified on the first plasmid belonging to IncFIB (JN420336) in KPCTRSRTH06 (Table 1).

In our study, none of the five *K. pneumoniae* isolates from patients had *mcr* genes, even though these isolates were highly resistant to colistin (≥ 32 mg/L) (Table 1). A detailed genomic interrogation of these genomes identified multiple mutations in the chromosomal genes, including *phoPQ, mgrB, pmrCAB, pmrE* and *arnBCADTEF* genes and intergenic regions that could potentially be associated with colistin resistance (Suppl. Table S4). Previously reported disruptions and mutations in *mgrB* [8, 40] and *pmrB* (D150Y) [41] conferring colistin resistance were observed in four of the five isolates: KPCTRPRTH01 (*mgrB*: Q30*), KPCTRPRTH02 (*pmrB*: D150Y), KPCTRPRTH03 (*mgrB*: W20*), and KPCTRPRTH04 (*pmrB*: D150Y) (Suppl. Table S4). In the two isolates with the *pmrB* (D150Y) mutation, KPCTRPRTH02 and KPCTRPRTH04, a *pmrA* pseudogene, affecting the transcriptional regulatory domain reported to increase colistin resistance [42], was also observed (Suppl. Table S4). In isolate KPCTRPRTH05, no previously identified mutations were detected. Instead, a novel S60L mutation in uncharacterized protein YcaR was identified. Located within the KDO2-lipid A biosynthesis gene cluster and co-transcribed with *kdsB* [43], *ycaR* could serve as a novel candidate gene that potentially influences colistin resistance. Additionally, an interrogation of 84 publicly available *Klebsiella* spp. genomes that share the same ST16 (*n* = 54) and ST231 (*n* = 30) profile as the Thailand isolates revealed only five genomes not isolated in Thailand with a similar *mgrB* disruption (ST16, *n* = 2; ST231, *n* = 3). All other identified mutations were unique to these STs, suggesting that new resistant mechanisms potentially have emerged in these lineages.

### Phylogenetic relationship of colistin-resistant *K. pneumoniae* isolates

**Fig. 2:**
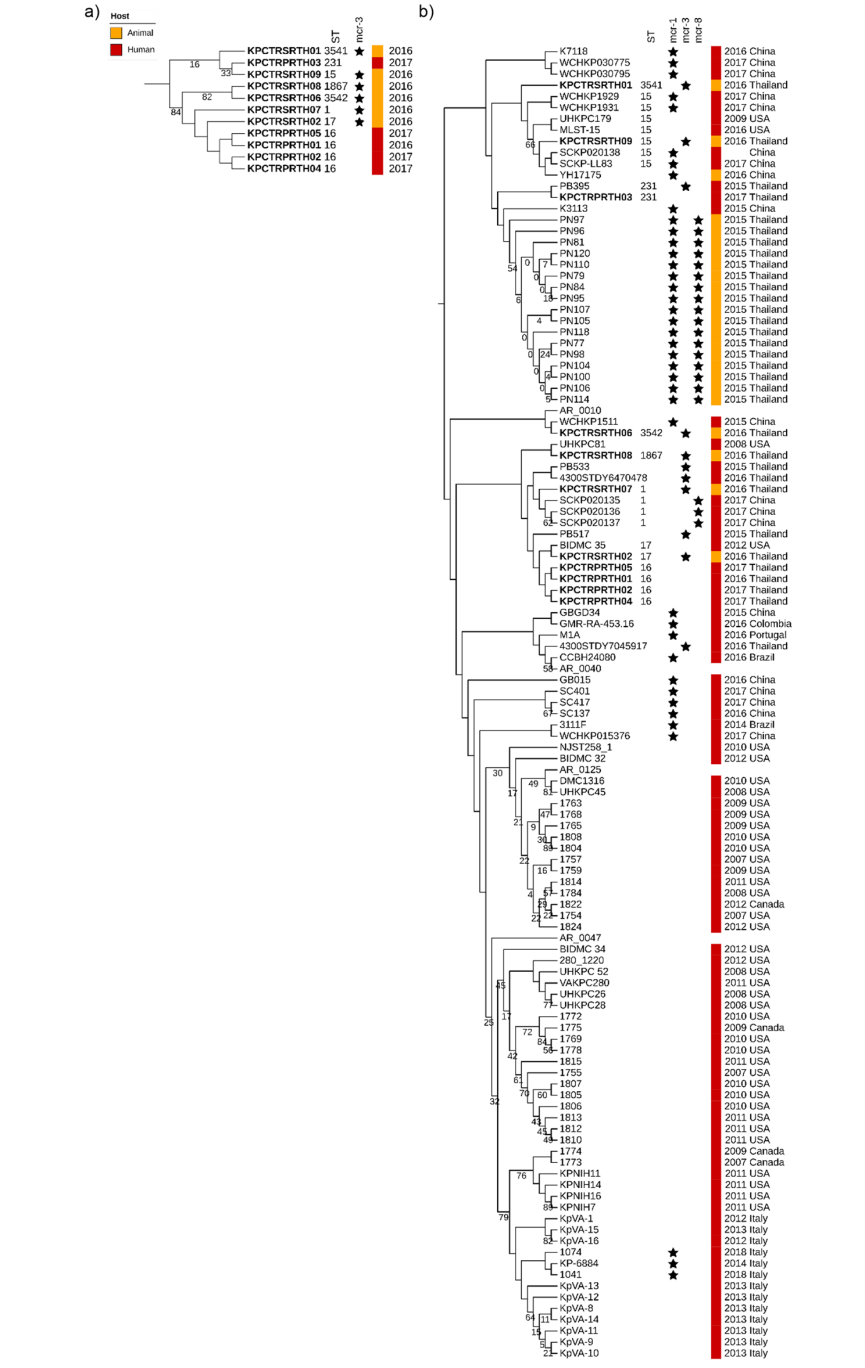
**Phylogenetic analysis of***K. pneumoniae***isolates from hospitalized patients and healthy swine in Thailand**. Maximum-likelihood phylogeny tree inferred from (**a**) 69,435 recombinant-filtered core SNPs of *K. pneumoniae* genomes from Thailand analyzed in this study (*n* = 11), using *K. pneumoniae* KPCTRSRTH02 as the reference. (**b**) 109,250 recombinant-filtered core SNPs of 117 colistin-resistant *K. pneumoniae* genomes identified in the GenBank database, using *K. pneumoniae* MLST-15 (CP022125-CP022128) as the reference. Midpoint rooted and branch lengths were ignored. The ST (only for isolates from Thailand), host, isolation year, and geographical origin of the isolates are displayed, where information was not available, or the gene was not present, it was left blank. The presence of colistin-resistance *mcr* genes is indicated by a star symbol. Thailand isolates from this study are indicated in bold. Numbers at nodes represent < 90% bootstrap support

Phylogenetic analysis of the eleven colistin-resistant *K. pneumoniae* isolates from this study revealed high genomic diversity, with only the ST16 isolates (*n* = 4) clustering together and the pairwise SNP distance between any two of the ST16 isolates averaging 0.018% or 918 SNP sites (range: 0.006% or 306 SNP sites to 0.032% or 1,633 SNP sites) (Fig. 2a). None of the *K. pneumoniae* isolates with *mcr*-3 gene variants from swine clustered together (Fig. 2a), confirming that they were not the result of clonal expansion. We further compared the 11 *K. pneumoniae* genomes from our dataset with 117 publicly available colistin-resistant *K. pneumoniae* genomes to investigate the geographical spread of *mcr*-positive isolates (Fig. 2b). Isolates from this study were more likely to cluster together with isolates previously reported from Thailand and China, as well as a few isolates from the USA (Fig. 2b). Phylogenetic analyses revealed that *mcr-*positive *K. pneumoniae* isolates were widely dispersed across the global *mcr*-positive *K. pneumoniae* isolate tree, indicating geographical spread across countries (Fig. 2b). Interestingly, we only detected the *mcr*-3 gene in *K. pneumoniae* isolates from swine, whereas the *mcr*-3 gene was detected in *E. coli* isolates from swine and humans (Table 1).

### Phylogenetic relationship of colistin-resistant *E. coli* isolates

**Fig. 3:**
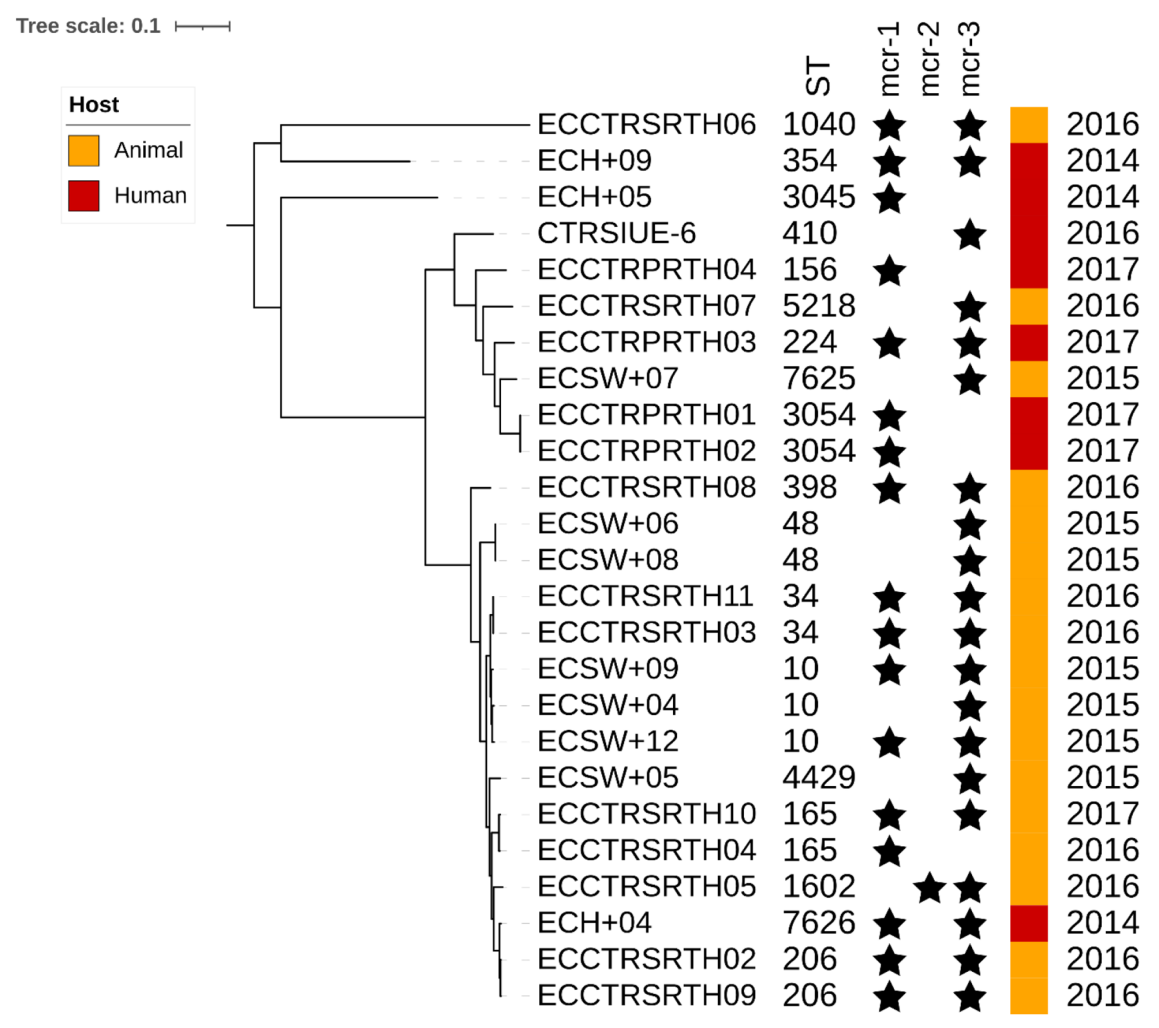
**Phylogenetic analysis of***E. coli***isolates from farmers, swine, and hospitalized patients in Thailand**. Maximum-likelihood phylogeny tree inferred from 160,485 recombinant-filtered core SNPs of *E. coli* genomes isolated in Thailand analyzed in this study, using *E. coli* ECSW + 09 as the reference. The ST, host, and isolation year are shown. The presence of colistin-resistance *mcr* genes is indicated by a star symbol, if the gene was not present the area was left blank. The scale bar represents the number of nucleotide substitutions per site. Numbers at nodes represent <90% bootstrap support

**Fig. 4:**
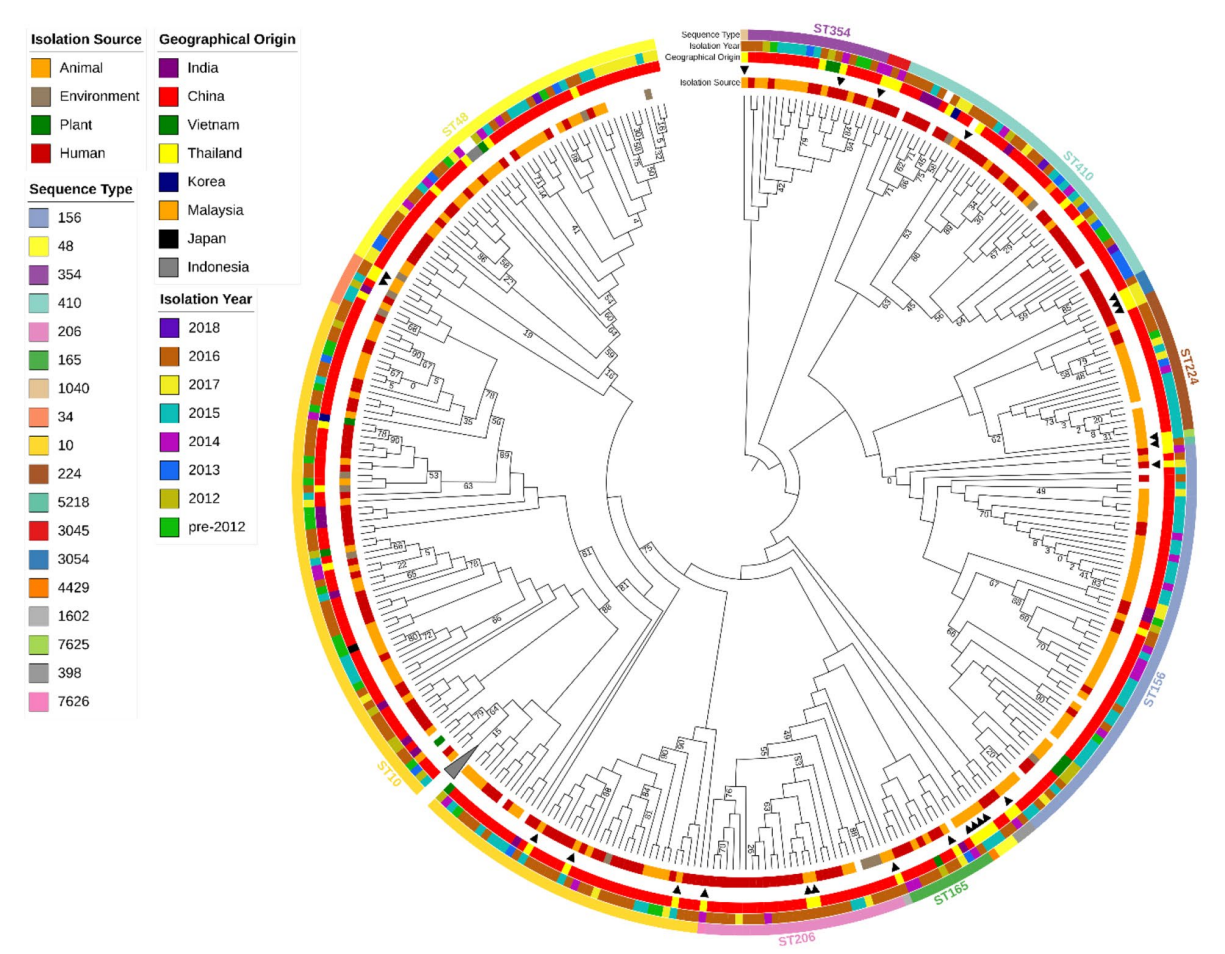
**Phylogenetic analysis of***E. coli***isolates from Asia**. Maximum-likelihood phylogeny inferred from 107,712 recombinant filtered core SNPs of 408 *E. coli* genomes isolated from Asia that shared the same ST as the isolates from Thailand (labelled with a black triangle) and 25 *E. coli* genomes from this study. *E. coli* strain K-12 substrain MG1655 (CP012868) was used as the reference. Midpoint rooted and branch lengths were ignored. The collapsed node represents 47 ST10 genomes from China and India. The host, isolation year, and geographical origin are displayed. Where information was not available it was left blank. Numbers at nodes represent < 90% bootstrap support

Phylogenetic analysis of the 25 *E. coli* isolates showed our isolates formed two major clades (Fig. 3). Interestingly, we found two isolates that were closely related, an isolate from a farmer (ECH + 04) and an isolate from swine (ECCTRSRTH09); both coharbored *mcr*-1.1 and *mcr*-3.1, even though these isolates were collected two years apart. Overall, two- to five-SNP differences were observed within these two genomes, which both coharbored *mcr*-1.1 and *mcr*-3.1 despite both isolates being collected 2 years apart. Our 25 *E. coli* genomes were compared with 408 publicly available colistin-resistant *E. coli* genomes to investigate the dissemination of colistin-resistant and *mcr*-positive isolates in Asia (Fig. 4). *E. coli* genomes from our study were distributed throughout and clustering with isolates from other countries, confirming that the other colistin-resistant strains similar to the ones described in our study were previously reported and suggesting spread across Asia (Fig. 4).

## Discussion

We observed a colonization rate of 26.2% for colistin-resistant Enterobacterales (39/149) in our study, with *E. coli* and *K. pneumoniae* being the primary bacterial species identified. The colonization rates of colistin-resistant *E. coli* isolates found in the present study were relatively high, especially among the swine isolates, where the rate of colonization was at 58.6%, compared to only ~ 3% colonization rate of colistin-resistant *E. coli* found in 400 swine from a northern and an eastern province of Thailand in 2012 [44]. However, the high rate in our study could be explained by a small number of swine (*n* = 38) sampled. The prevalence of colistin-resistant isolates among farmers was 10.7%, though previous study has found no colistin-resistant isolates carriage among 30 farmers in 2012 study [44]. In the international literature, only few studies identified carriage in farmers, and reported that the prevalence of colistin-resistant isolates among farmers was < 5% [45, 46]. In contrast, the prevalence of colistin-resistant Enterobacterales among our hospitalized patients (12.1%) was not significantly different from the 17.1% prevalence reported in the most recent study conducted in 2018 in Thailand [47].

We identified *E. coli* with a new *mcr*-2 gene variant, *mcr-2.3*, among the swine isolates. Typically, *mcr* genes are found on plasmids; however, co-occurrence of genes on both plasmid and chromosome has been rarely discovered [48–50]. In our study, *E. coli* harbored *mcr*-2.3 on the chromosome rather than plasmid, suggesting that *mcr*-2.3 could have alternative pathways to get into bacterial strains. Additionally, one *K. quasipneumoniae* subsp. *similipneumoniae* isolate from swine was found to harbor two copies *mcr*-3.21, one on its plasmid and one on the chromosome, while the other three isolates carried *mcr*-3.21 on plasmids only. The discovery of an isolate with *mcr* on both its plasmid and chromosome suggests that the process for stabilizing the *mcr*-3.21 gene within the bacterial genome is still in progress. Although several studies of *E. coli* have shown the chromosomal location of *mcr*-1 and *mcr*-2 [50, 51], the finding of *mcr*-3.21 on both a plasmid and chromosome in a single isolate has recently been reported in healthy individuals, farmers and swine in Thailand [48, 49]. This finding was possible due to opportunity to do long- and short- read sequencing on some of the isolates, and hybrid assembly of these reads allowed a more closed genomes to be analyzed. The presence of these *mcr* variants on chromosome is concerning because it has high potential to spread *mcr* genes due to vertical and horizontal gene transfer. Notably, *E. coli* and *K. quasipneumoniae* are highly prevalent in swine and other livestock, where they could interact with organisms from different hosts [52], which facilitates the exchange of antimicrobial resistance carrying plasmids such as *bla*_CTX−M_ on IncF plasmid as has been shown in study in China [53, 54], suggesting that this could happen with *mcr*-carrying plasmids as well.

The present study detected plasmids harboring *mcr*-1.1 and variants of *mcr*-3 separately or in combination in *E. coli* isolated from swine and farmers in Thailand. The co-occurrence of two *mcr* genes in a single bacterial isolate is intriguing. A few reports have described the co-occurrence of *mcr*-1 and *mcr*-3 on a single plasmid of *E. coli* [55, 56]. Interestingly, genes *mcr*-4 to *mcr*-10 were not identified in our study. According to previous reports, *mcr*-4 and *mcr*-5 have been detected in *E. coli* from Asia and European countries, whereas *mcr*-7.1 and *mcr*-8 have been detected in *K. pneumoniae* from China [57–59]. The finding of *mcr* and *bla* genes in swine isolates demonstrates the effects of amoxicillin and colistin use in pig farms in northern Thailand. Additionally, a recent study revealed that amoxicillin and colistin were also used in some small-to-medium scale pig farms in northeastern Thailand [6].

In a recent study on pig farms in Thailand, 4.5% (*n* = 31/696) of pathogenic *E. coli* harbored *mcr*-1, and these *mcr* genes were located on IncF-type [60]. Such findings relating to plasmid incompatibility groups were similar to those of our study. Regarding plasmid replicon typing, the host range of the IncF plasmids is limited to Enterobacterales, and IncF plasmids have been detected in Enterobacterales globally, associated with various antimicrobial-resistant genes [61]. IncX4-type plasmids are now considered vehicles responsible for the dissemination of the *mcr*-1 gene among Enterobacterales worldwide [62–64]. The IncX4 plasmid architecture has been reported to be highly conserved, with several studies having shown similar IncX4 plasmids carrying *mcr*-1 from different Enterobacterales [62, 63]. In the present study, we report *E. coli* isolates carrying *mcr*-1.1 on IncX4 plasmids from both hospitalized patients and swine. This is a significant finding because this rare plasmid has been identified in a small dataset. However, further studies involving larger datasets are now required to confirm the prevalence of this plasmid. The presence of the IncX4 plasmid was verified by long-read sequencing using MinION, which provides confidence in the results. Among the *Klebsiella* spp. identified in this study, IncFII (pMET) was the most common replicon type identified among isolates from swine. IncFII is a plasmid with a narrow host range that is frequently identified among *E. coli* strains carrying the *mcr*-1 gene isolated from swine worldwide [65]. Recently, IncFII was identified in Thailand and Laos along with the emergence of *mcr*-3-mediated plasmids such as IncP1 and IncI1 [66, 67]. The finding of IncFII (pMET) in *Klebsiella* spp. supports the notion that *mcr*-3 can potentially spread among different Enterobacterales and disseminate to neighboring countries.

We did not detect mobile colistin resistance genes in any of the patient isolates. However, the mutations in *mgrB* or *pmrAB* observed in four of five patient isolates were consistent with previous studies, suggesting that the spread of colistin-resistant *K. pneumoniae* in hospitals is most likely due to chromosomal mutation [68–70]. Interestingly, the colistin-resistant isolates with chromosomal mutations had higher MICs than the *mcr*-positive isolates, making clinical treatment more difficult. Moreover, a *pmrA* pseudogene was found in the isolates containing the mutated *pmrB* gene. Although the *pmrA* pseudogene causes an increase in the level of polymyxin B susceptibility by reducing cationic groups on the LPS [19], the isolates in our study containing both the *pmrA* pseudogene and the altered *pmrB* gene had a high level of colistin resistance, suggesting that the loss of *pmrA* gene function may have promoted a different lipid A modification pathway. We also identified a novel mutation in protein YcaR, which is part of the KDO2–lipid A biosynthetic pathway [43], in the *K. pneumoniae* isolates from hospitalized patients. However, further genomic analyses of this point mutation are required to determine whether this mutation is present in other colistin-resistant strains without the *mcr* gene.

There are several limitations to our study. First, direct horizontal transmission between swine and farmers could not be demonstrated because of the limited number of colistin-resistant isolates and the lack of longitudinal sampling. However, despite the study’s relatively small sample size, we were still able to demonstrate the presence of *bla* and colistin-resistance genes related to the antibiotics used in swine production in Thailand. Second, the fecal samples from farmers and hospital patients were collected from individuals in different provinces; consequently, it might be difficult to demonstrate linkage among bacterial clones between two provinces. However, there was similarity in the *mcr* gene types detected among the *E. coli* isolates from both locations.

## Conclusion

Our study demonstrates that bacterial isolates from farmers and swine in Thailand contained multiple antimicrobial-resistance genes, such as extended spectrum beta-lactamases genes and colistin-resistance genes. Even though the present study cannot directly demonstrate horizontal transmission of bacterial strains or plasmid-mediated colistin-resistance genes between swine, farmers, and hospitalized patients, plasmid analysis revealed that the mobile colistin-resistance *mcr*-1.1 genes of isolates from swine and hospitalized patients belonged to the same incompatibility group. Our findings confirmed that *mcr*-positive *E. coli* isolates may be endemic in Thailand, additionally, the highly conserved IncX4 plasmids might be becoming endemic too. Notably, when we compared colistin-resistant Enterobacterales isolates from animals and farmers to the colistin-resistant Enterobacterales isolates from hospitalized patients at Siriraj Hospital, the main mechanism of colistin resistance was a chromosomal point mutation. While our study focused on plasmid-mediated *mcr* genes, our findings also highlight the emergence of colistin resistance mediated by specific mutations in *mgrB* or *pmrAB* present on the chromosome. The latter is important with respect to hospitalized patients because the chromosomal mutations usually confer a high level of colistin resistance (MIC ≥ 32).

### Electronic supplementary material

Below is the link to the electronic supplementary material.

Supplementary Material 1

## Data Availability

Individual level data cannot be shared publicly because of patient confidentiality under current Thai legislation. The data that support the findings of this study are available from Faculty of Medicine Siriraj Hospital, Mahidol University, Thailand, but restrictions apply to the availability of these data, which were used under license for the current study, and so are not publicly available. Data are however available from Dr. Adhiratha Boonyasiri (Email: adhiratha.bon@mahidol.ac.th) upon reasonable request and with permission of Faculty of Medicine Siriraj Hospital, Mahidol University, Thailand and the appropriate ethics committee. All sequencing reads were deposited in the SRA database under the project accession number PRJNA389557, https://www.ncbi.nlm.nih.gov/bioproject/?term=PRJNA389557. Relevant assemblies and *mcr*-genes have been deposited in GenBank under accession numbers as shown in Suppl. Tables S1 and S2).
