# Supplementary material for: Characteristics and genomic epidemiology of colistin-resistant Enterobacterales from farmers, swine, and hospitalized patients in Thailand, 2014–2017
Source: BMC Infect Dis. 2023 Aug 28;23:556. doi: 10.1186/s12879-023-08539-8 (PMC10464208; doi:10.1186/s12879-023-08539-8)
Supplement: Supplementary file 1 — Supplementary Material 1 [file 12879_2023_8539_MOESM1_ESM.docx]

**SUPPLEMENTARY MATERIALS**

*Sequencing and assembly*

Short-read genome sequencing was performed on the 39 Thailand isolates. The resulting average *de novo* assembly sequence coverage was 138-fold (*E. coli*) and 133-fold (*Klebsiella* spp.) across an average number of contigs per genus (164 for *Escherichia* 98 for *Klebsiella*) resulting in average draft genome sizes of 5.0 and 5.5 Mbp for *E. coli* and *Klebsiella* spp. isolates respectively. Oxford Nanopore MinION sequencing was performed on a subset of 12 *mcr*-positive isolates to improve the respective draft genome assemblies. The resulting average MinION sequence coverage was 18-fold (*E. coli*) and 21-fold (*Klebsiella* spp.), with hybrid assemblies yielding fewer average number of contigs per genus (57 for *Escherichia*; 22 for *Klebsiella*) (**Supplementary Table S1 and S2**).

**Table S1.** Accessions and assembly statistics of Thailand isolates analysed as part of this study.

| Strain | Biosample  no. | Accession no. | Organism | Number of contigs | Length (Mbp) | Contig N50 | Coverage |
| --- | --- | --- | --- | --- | --- | --- | --- |
| CTRSIUE-6  (ECCTRPUTH01) | SAMN07203027 | GCA_002290415.1 | *E. coli* | 175 | 5.2 | 148987 | 81.33x |
| ECCTRPRTH01 | SAMN07450765 | GCA_002246755.1 | *E. coli* | 108 | 5 | 197375 | 147.11x |
| ECCTRPRTH02 | SAMN07450580 | GCA_002246495.2 | *E. coli* | 68 | 6.2 | 14978 | 142.21x |
| ECCTRPRTH03 | SAMN07450579 | GCA_002246485.2 | *E. coli* | 64 | 6.9 | 50117 | 162.63x |
| ECCTRPRTH04 | SAMN07450578 | GCA_002246515.1 | *E. coli* | 163 | 5.1 | 102282 | 149.93x |
| ECCTRSRTH02 | SAMN07450617 | GCA_002246085.1 | *E. coli* | 189 | 5.1 | 68358 | 138.15x |
| ECCTRSRTH03 | SAMN07450616 | GCA_002246595.3 | *E. coli* | 12 | 6.2 | 58201 | 150.12x |
| ECCTRSRTH04 | SAMN07450615 | GCA_002246115.2 | *E. coli* | 180 | 6.7 | 4312 | 129.63x |
| ECCTRSRTH05 | SAMN07450614 | GCA_002247905.2 | *E. coli* | 19 | 6.2 | 59029 | 156.89x |
| ECCTRSRTH06 | SAMN07450613 | GCA_002247965.2 | *E. coli* | 35 | 6.5 | 59096 | 160.21x |
| ECCTRSRTH07 | SAMN07450612 | GCA_002247975.2 | *E. coli* | 17 | 6.8 | 61812 | 141.15x |
| ECCTRSRTH08 | SAMN07450611 | GCA_002251815.2 | *E. coli* | 21 | 6.2 | 59163 | 148.16x |
| ECCTRSRTH09 | SAMN07450610 | GCA_002247995.2 | *E. coli* | 14 | 6.1 | 58286 | 155.28x |
| ECCTRSRTH10 | SAMN07450609 | GCA_002248015.1 | *E. coli* | 148 | 4.9 | 108996 | 144.46x |
| ECCTRSRTH11 | SAMN07450713 | GCA_002249645.1 | *E. coli* | 152 | 4.9 | 108769 | 194.04x |
| ECH+04 | SAMN07450679 | GCA_002248805.1 | *E. coli* | 170 | 5 | 73950 | 140.93x |
| ECH+05 | SAMN07450678 | GCA_002247945.1 | *E. coli* | 136 | 5.2 | 110528 | 134.4x |
| ECH+09 | SAMN07450674 | GCA_002247425.1 | *E. coli* | 104 | 5.2 | 205166 | 157.39x |
| ECSW+04 | SAMN07450635 | GCA_002247695.1 | *E. coli* | 262 | 5.5 | 75413 | 132.35x |
| ECSW+05 | SAMN07450634 | GCA_002247735.1 | *E. coli* | 198 | 5.1 | 108206 | 118.87x |
| ECSW+06 | SAMN07450633 | GCA_002247725.1 | *E. coli* | 152 | 4.8 | 78391 | 124.7x |
| ECSW+07 | SAMN07450632 | GCA_002247775.2 | *E. coli* | 29 | 6.2 | 31771 | 129.95x |
| ECSW+08 | SAMN07450631 | GCA_002247765.2 | *E. coli* | 35 | 6.2 | 14119 | 152.17x |
| ECSW+09 | SAMN07450630 | GCA_002247815.1 | *E. coli* | 152 | 4.9 | 93896 | 132.43x |
| ECSW+12 | SAMN07450627 | GCA_002248785.1 | *E. coli* | 192 | 4.8 | 75775 | 140.26x |
| KPCTRPRTH01 | SAMN07450577 | GCA_002246875.1 | *K. pneumoniae* | 90 | 5.6 | 237653 | 128.22x |
| KPCTRPRTH02 | SAMN07450622 | GCA_002246555.1 | *K. pneumoniae* | 87 | 5.6 | 194943 | 120.89x |
| KPCTRPRTH03 | SAMN07450621 | GCA_002246765.1 | *K. pneumoniae* | 129 | 5.5 | 142900 | 148.02x |
| KPCTRPRTH04 | SAMN07450620 | GCA_002246035.1 | *K. pneumoniae* | 88 | 5.6 | 194943 | 146.21x |
| KPCTRPRTH05 | SAMN07450619 | GCA_002246045.1 | *K. pneumoniae* | 96 | 5.6 | 206016 | 135.53x |
| KPCTRSRTH01 | SAMN07450608 | GCA_002251875.3 | *K. pneumoniae* | 7 | 7.2 | 65196 | 161.03x |
| KPCTRSRTH02 | SAMN07450607 | GCA_002248045.2 | *K. pneumoniae* | 14 | 7.1 | 66007 | 125.46x |
| KPCTRSRTH03 | SAMN07450606 | GCA_002248055.2 | *K. quasipneumoniae subsp. similipneumoniae* | 22 | 7.2 | 65095 | 128.62x |
| KPCTRSRTH04 | SAMN07450605 | GCA_002248085.2 | *K. quasipneumoniae subsp. similipneumoniae* | 63 | 7.4 | 29010 | 109.1x |
| KPCTRSRTH05 | SAMN07450604 | GCA_002248945.2 | *K. quasipneumoniae subsp.similipneumoniae* | 11 | 6.9 | 63858 | 122.18x |
| KPCTRSRTH06 | SAMN07450603 | GCA_002248105.2 | *K. pneumoniae* | 12 | 7.2 | 65486 | 151.3x |
| KPCTRSRTH07 | SAMN07450602 | GCA_002248115.3 | *K. pneumoniae* | 11 | 7.3 | 65728 | 140.66x |
| KPCTRSRTH08 | SAMN07450601 | GCA_002248135.1 | *K. pneumoniae* | 96 | 5.4 | 195443 | 118.55x |
| KPCTRSRTH09 | SAMN07450600 | GCA_002248955.2 | *K. pneumoniae* | 11 | 6.8 | 65525 | 162.46x |

Number of contigs with contig length greater than 500bp; Contig N50: length such that sequence contigs of this length or longer include half the bases of the assembly; Contig L50 - number of sequence contigs that are longer than, or equal to, the N50 length and therefore include half the bases of the assembly.

**Table S2.** Accessions of *mcr*-containing Thailand complete plasmids analysed as part of this study.

| Strain | Biosample  no. | Accession no. | Organism | Length (bp) |
| --- | --- | --- | --- | --- |
| ECCTRPRTH02 | SAMN07450580 | CM016890.1 | *E. coli* | 33,836 |
| ECCTRPRTH03 | SAMN07450579 | CM016891.1 | *E. coli* | 74,015 |
| ECCTRSRTH03 | SAMN07450616 | CP041113.1 | *E. coli* | 63,011 |
|  |  | CP041114.1 |  | 58,983 |
| ECCTRSRTH05 | SAMN07450614 | CM016892.1 | *E. coli* | 133,016 |
| ECCTRSRTH08 | SAMN07450611 | CM016893.1 | *E. coli* | 33,836 |
| ECCTRSRTH09 | SAMN07450610 | CM016894.1 | *E. coli* | 101,345 |
|  |  | CM016895.1 |  | 35,075 |
| KPCTRSRTH01 | SAMN07450608 | CP041095.1 | *K. pneumoniae* | 70,501 |
| KPCTRSRTH04 | SAMN07450605 | CM016900.1 | *K. quasipneumoniae subsp. similipneumoniae* | 34,774 |
| KPCTRSRTH05 | SAMN07450604 | CM016896.1 | *K. quasipneumoniae subsp. similipneumoniae* | 71,655 |
| KPCTRSRTH06 | SAMN07450603 | CM016897.1 | *K. pneumoniae* | 236,458 |
|  |  | CM016898.1 |  | 35,685 |
| KPCTRSRTH07 | SAMN07450602 | CP041104.1 | *K. pneumoniae* | 36,884 |
| KPCTRSRTH09 | SAMN07450600 | CM016899.1 | *K. pneumoniae* | 35,685 |

**Figure S1.** Alignment of protein sequences of MCR-2.1 (SBV31106) with MCR-2.3^*^(NG_065452). Alignments were generate in Clustal Omega v 1.2.4 (1) and were presented using Jalview v2.11.2.0 (2).


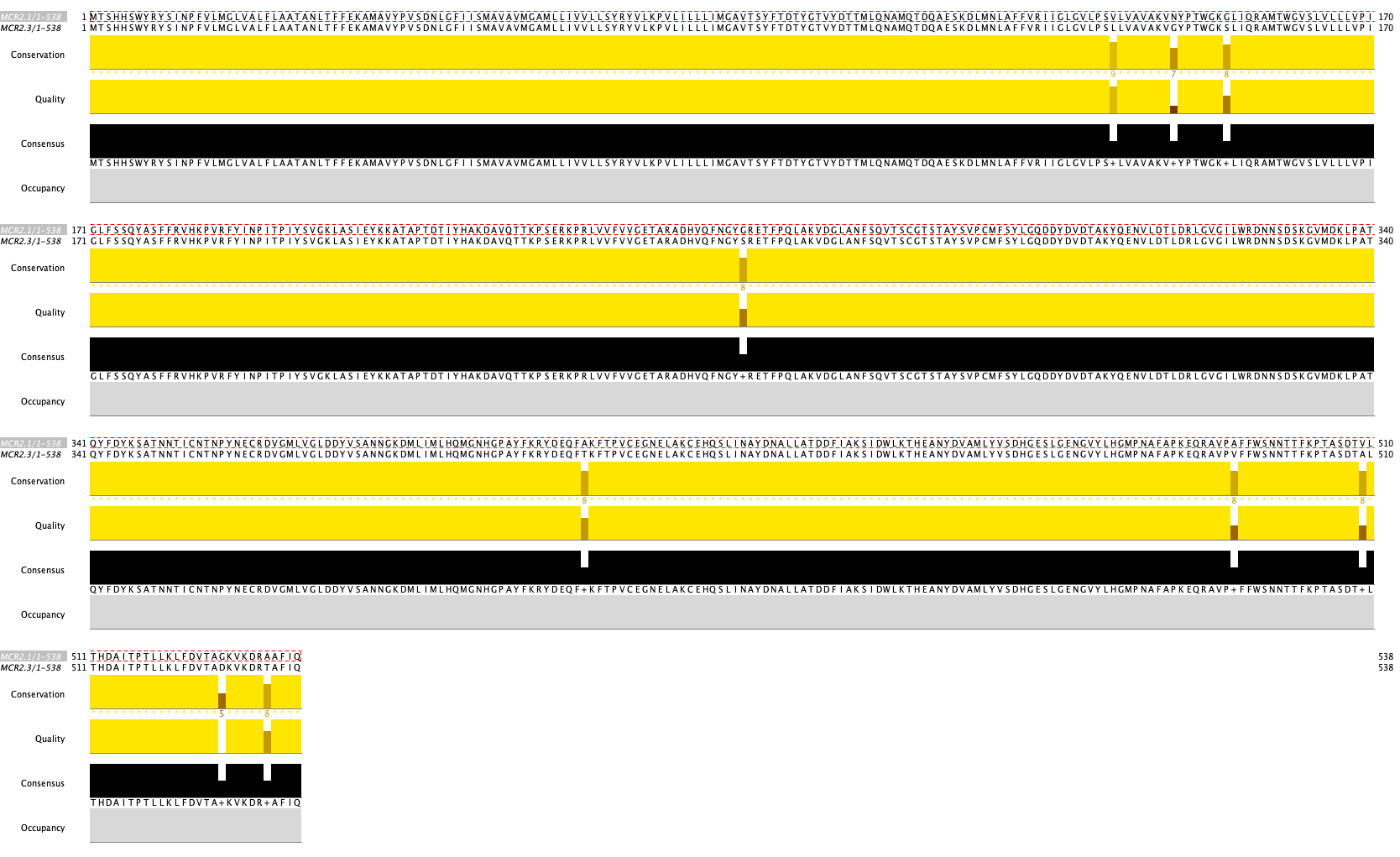


**Figure S2.** Alignment of protein sequences of MCR-3.1(ASF81896) with MCR-3.21 (QDJ85872), MCR-3.22 (QDJ80325), MCR-3.23 (NG_060583) and MCR-3.24 (NG_060580). Alignments were generate in Clustal Omega v 1.2.4 (1) and were presented using Jalview v2.11.2.0 (2).


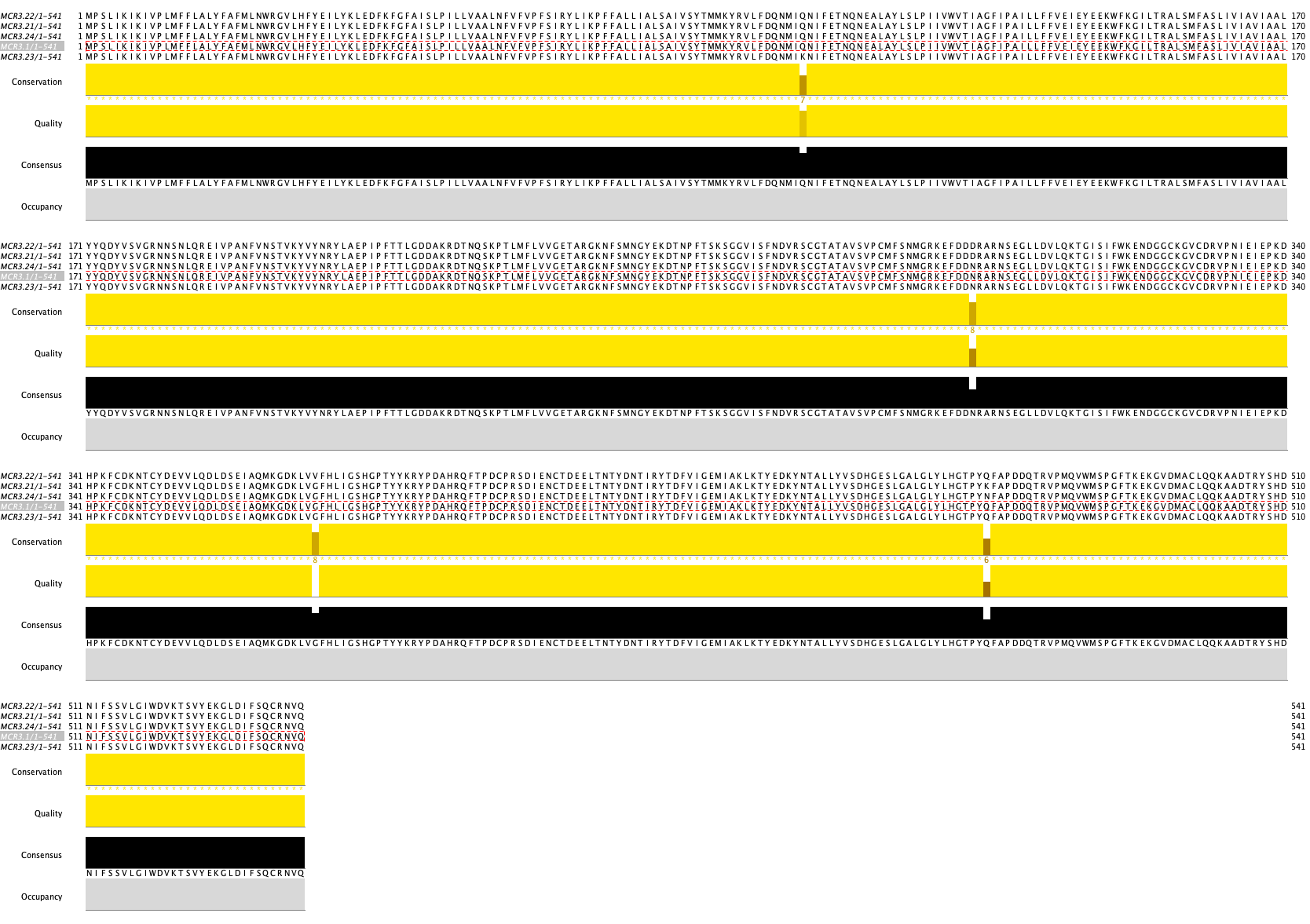


**Table S3.** Comparison of novel MCR variants to MCR-2.1(SBV31106) and MCR-3.1(ASF81896).

| **Strain** | **Allele (Accession no.)** | **Comparison to MCR-2.1**^*^ **and MCR-3.1**^†^ | |
| --- | --- | --- | --- |
|  |  | **% Identity** | **Peptide change** |
| ECCTRSRTH05 | MCR-2.3^*^  (NG_065452) | 98.33% | L136V, G144N, S151G, S257G, T406A, V492A, A509V, D528G, T534A |
| KPCTRSRTH04, KPCTRSRTH06, KPCTRSRTH07, KPCTRSRTH09 | MCR-3.21^†^  (QDJ85872) | 99.63% | N296D, Q468K |
| KPCTRSRTH01, KPCTRSRTH02, KPCTRSRTH03, KPCTRSRTH05 | MCR-3.22^†^  (QDJ80325) | 99.63% | N296D, G373V |
| KPCTRSRTH08 | MCR-3.23^†^  (NG_060583) | 99.82% | Q102K |
| ECSW+12 | MCR-3.24^†^  (NG_060580) | 99.82% | Q468N |

**Table S4.** Chromosomal and intergenic mutations unique to *mcr* negative Thailand isolates.

| KPCTRPRTH01 | KPCTRPRTH02 | KPCTRPRTH04 | KPCTRPRTH05 | KPCTRPRTH03 | Nucleotide Change^†^ | Peptide Change^†^ | Position^†^ | Locus Name^†^ | Protein Product^†^ |
| --- | --- | --- | --- | --- | --- | --- | --- | --- | --- |
| T | T | G | T | T | - | - | 5296859 | - | - |
| G | T | G | G | G | tCg/tAg | S220* | 653965 | KPN_RS03140 | ABC transporter permease |
| A | T | T | A | A | aTa/aAa | I72K | 261741 | KPN_RS01210 | hydroxyacylglutathione hydrolase |
| T | A | A | T | T | cAg/cTg | Q91L | 326476 | KPN_RS01545 | phosphonate ABC transporter, permease protein PhnE |
| G | A | A | G | G | tGg/tAg | W81* | 645932 | KPN_RS03095 | amino acid ABC transporter substrate-binding protein |
| C | A | A | C | C | Gac/Tac | D150Y | 901908 | KPN_RS04350 | two-component system sensor histidine kinase PmrB |
| Absent | Present | Present | Absent | Absent | 10bpΔ | T151Sfs |  | KPN_RS04355 | two-component system sensor histidine kinase PmrA (*pmr*A pseudogene) |
| C | T | T | C | C | Gag/Aag | E74K | 1091807 | KPN_RS05170 | Lrp/AsnC family transcriptional regulator |
| G | A | A | G | G | gCc/gTc | A204V | 1168178 | KPN_RS05455 | aldehyde dehydrogenase PuuC |
| G | A | A | G | G | Gcc/Acc | A260T | 1416306 | KPN_RS06750 | transcriptional regulator GcvA |
| A | G | G | A | A | gTc/gCc | V438A | 1740425 | KPN_RS08390 | malonate decarboxylase subunit alpha |
| A | T | T | A | A | gAa/gTa | E35V | 1950088 | KPN_RS09505 | oxamate amidohydrolase |
| C | A | A | C | C | gGc/gTc | G411V | 2343882 | KPN_RS11535 | Fe-S cluster assembly protein SufB |
| C | T | T | C | C | gGg/gAg | G80E | 2351753 | KPN_RS11565 | efflux RND transporter periplasmic adaptor subunit |
| C | C | C | C | T | tgG/tgA | W20* |  | KPN_RS29045 | PhoP/PhoQ regulator MgrB |
| A | G | G | G | G | Cag/Tag | Q30* |  | KPN_RS29045 | PhoP/PhoQ regulator MgrB |
| C | G | G | C | C | Gcc/Ccc | A205P | 2678631 | KPN_RS13240 | GGDEF-domain containing protein |
| G | A | A | G | G | Ccc/Tcc | P52S | 2992110 | KPN_RS14605 | phosphohistidine phosphatase SixA |
| G | A | A | G | G | - | - | 3582372 | - | - |
| G | A | A | G | G | aCt/aTt | T181I | 4047691 | KPN_RS19940 | DNA-protecting protein DprA |
| G | A | A | G | G | Gtg/Atg | V354M | 4397280 | KPN_RS21650 | xanthine permease |
| T | C | C | T | T | Acg/Gcg | T69A | 4525675 | KPN_RS22280 | phosphate ABC transporter, permease protein PstA |
| A | T | T | A | A | Aaa/Taa | K11* | 5193640 | KPN_RS25560 | DNA-binding response regulator |
| T | T | T | T | G | - | - | 2338311 | - | - |
| A | A | A | A | G | - | - | 3515867 | - | - |
| A | A | A | A | T | - | - | 3515890 | - | - |
| C | C | C | C | T | tCt/tTt | S917F | 4772826 | KPN_RS23500 | DNA-directed RNA polymerase subunit beta |
| A | G | G | G | G | Gcc/Acc | A330T | 220219 | KPN_RS01005 | 1-deoxy-D-xylulose-5-phosphate reductoisomerase |
| A | C | C | C | C | - | - | 1676361 | - | - |
| A | C | C | C | C | - | - | 1676367 | - | - |
| A | C | C | C | C | - | - | 2299028 | - | - |
| G | C | C | C | C | gGg/gCg | G339A | 2341873 | KPN_RS11525 | signal peptide peptidase SppA |
| A | C | C | C | C | cGc/cTc | R77L | 2458332 | KPN_RS12080 | membrane protein |
| T | G | G | G | G | ttC/ttA | F177L | 2527147 | KPN_RS12425 | murein transglycosylase |
| A | G | G | G | G | Ggc/Agc | G252S | 2706166 | KPN_RS13375 | imidazole glycerol phosphate synthase cyclase subunit |
| T | C | C | C | C | - | - | 2747346 | - | - |
| C | T | T | T | T | gAt/gGt | D236G | 2786563 | KPN_RS13670 | xylulokinase |
| T | C | C | C | C | Ccg/Tcg | P49S | 3888172 | KPN_RS19085 | DedA family protein |
| T | C | C | C | C | aCc/aTc | T573I | 4775899 | KPN_RS23505 | DNA-directed RNA polymerase subunit beta' |
| C | C | C | T | C | tCa/tTa | S60L | 1065294 | KPN_RS05080 | protein YcaR |
| C | C | C | A | C | Gaa/Taa | E377* | 1342763 | KPN_RS06385 | PrkA family serine protein kinase |
| A | A | A | T | A | aaT/aaA | N68K | 2199948 | KPN_RS28955 | hypothetical protein |
| G | G | G | C | G | tgG/tgC | W13C | 3891496 | KPN_RS19120 | glutathione S-transferase family protein |
| A | A | A | G | A | - | - | 4891495 | - | - |

*, stop codon; fs, frameshift; -, not applicable, mutation fell in the intergenic region; ^†^ refers to *K*. *pneumoniae* MGH 78578 (CP000647-CP000652).

**References**

1. Sievers F, Wilm A, Dineen D, Gibson TJ, Karplus K, Li W, et al. Fast, scalable generation of high‐quality protein multiple sequence alignments using Clustal Omega. Molecular systems biology. 2011;7(1):539.

2. Waterhouse AM, Procter JB, Martin DM, Clamp M, Barton GJ. Jalview Version 2—a multiple sequence alignment editor and analysis workbench. Bioinformatics. 2009;25(9):1189-91.
